# Lung Cancer in Non-Smokers: Clinicopathological and Survival Differences from Smokers

**DOI:** 10.7759/cureus.32417

**Published:** 2022-12-11

**Authors:** Smreti Vasudevan, Vidya Krishna, Anurag Mehta

**Affiliations:** 1 Department of Research, Rajiv Gandhi Cancer Institute and Research Centre, Delhi, IND; 2 Department of Laboratory and Transfusion Services and Department of Research, Rajiv Gandhi Cancer Institute and Research Centre, Delhi, IND

**Keywords:** survival, adenocarcinoma, driver gene mutation, never smokers, lung cancer

## Abstract

Background

Lung cancer in non-smokers is a clinically distinct entity based on unique epidemiology, clinicopathology, genetics, treatment response, and outcome. Data from Indian centres are scarce. The objective of this study was to compare the frequency, clinical characteristics, driver mutations, and survival of non-smoking and smoking lung cancer patients treated at a tertiary cancer centre in North India.

Methodology

Two years of data on 724 consecutive lung cancer patients were assessed. Clinical, demographics, smoking history, and EGFR and ALK mutation test results were collected. Descriptive and inferential statistics were applied. Survival analysis was performed using the Kaplan-Meier method.

Results

Non-smokers comprised 40.9% of the study sample. Non-smokers were more likely than smokers to experience disease onset at a younger age (P = 0.004) and metastasis (P < 0.001). The tumor histology showed significant differences (P < 0.001), with non-smokers more likely to be diagnosed with adenocarcinoma (77.4%), while squamous and small cell histologies were commonly found among smokers (37.6% and 13.8%, respectively). The EGFR mutation and ALK rearrangement rates in the cohort were 23.3% and 10.1%, respectively, and were more frequent in non-smoking patients. Overall, 10-year survival was 7%, with a significantly better survival rate of non-smokers than smokers (median survival time of 15.13 vs 10.17 months; P = 0.012).

Conclusions

About four out of 10 patients diagnosed with lung cancer at our centre were non-smokers. They were more often young, diagnosed at an advanced stage, with predominantly adenocarcinoma histology, and had a threefold higher frequency of EGFR mutations than smokers. In our cohort, non-smokers appear to be a targetable group with better survival than smokers.

## Introduction

Globally, lung cancer is the second most common cancer, with 2.2 million cases and accounting for 1.8 million deaths [1]. In India, it is the fourth most common cancer, with 72,510 new cases and a high mortality rate, leading to 66,279 deaths in 2020 [1]. The number of new lung cancer cases in India is predicted to rise to 1,11,328 by the year 2025 [2].

Tobacco smoking is a major risk factor for lung cancer. However, the occurrence of lung cancer in patients with no smoking history has been increasing, and currently, 10-30% of lung cancer cases occur among non-smokers [3]. The percentage appears to be high in Asia, where non-smoking lung cancer is associated with onset at a young age, advanced stage, adenocarcinoma histology, female gender, and specific EGFR/ ALK/ ROS-1 gene alterations that can be treated with targeted therapeutics [4-9].

There is a paucity of literature from North India on clinicopathology and the survival of lung cancer patients based on smoking habits. Various published epidemiological studies comparing survival differences among smoking and non-smoking lung cancer patients have offered conflicting findings. While some studies suggest a better survival rate among non-smokers [10,11], other studies have indicated no survival difference [12-15]. Most of the studies have been conducted with a small sample size or short follow-up durations that fail to appreciate the differences. This study aims to evaluate the frequency, clinicopathological characteristics, and survival of smoking lung cancer patients in comparison with non-smoking lung cancer patients who received treatment at our centre.

## Materials and methods

Research setting, participants, and treatment

The treatment centre is a tertiary cancer hospital in North India that provides healthcare services to both urban and rural patients belonging to different parts of India. A total of 1,429 lung cancer cases were registered between January 2012 and April 2014. Of these, 754 patients underwent complete treatment at the centre. Depending on the extent of the disease, the patients in the sample were treated according to the standards of care, which consisted of surgery, chemotherapy, or radiotherapy. Chemotherapy medications included pemetrexed and cisplatin or gemcitabine and carboplatin. Advanced cases were offered tyrosine kinase inhibitors: Gefitinib/ Erlotinib/ Afatanib (for EGFR mutant cases) or Crizotinib (for ALK-rearranged cases), or immunotherapy (Nivolumab) at the physician's discretion.

Methodology

The study was approved by the Institutional Review Board (RGCIRC/IRB-BHR/119/2021, dated 30 June 2021) and conducted in compliance with the Declaration of Helsinki.

The electronic database was searched for medical records of lung cancer patients consecutively diagnosed between January 2012 and April 2014. The inclusion criteria were a confirmed histopathological diagnosis of lung cancer and having received treatment at the centre. Dual malignancy cases and patients with metastatic lesions from other organs to the lung were excluded from the study. Information pertaining to age, gender, region of residence in India, occupation, smoking history, presenting symptoms, stage of disease at presentation (AJCC, 7th edition guidelines) [16], histopathology, disease metastasis, co-morbidities, treatment history, and EGFR/ALK mutation test results were captured. The molecular testing for EGFR mutation was performed using quantitative real-time PCR, and ALK translocation was assessed by immunohistochemistry.

Non-smokers were defined as those who have smoked fewer than 100 cigarettes in their lifetime, and smokers were those who had a smoking history of at least 100 cigarettes in their lifetime [17]. The survival data was collected until the end of September 2022 by checking hospital follow-up visits or by telephonic contact.

Statistical analysis

The data was analysed using descriptive statistics. Categorical variables were presented as frequencies and percentages. To determine associations between categorical variables, Pearson's chi-squared test or Fisher's exact test was conducted. The Mann-Whitney test was performed to compare the median ages of the smoking and non-smoking groups. Overall, survival was defined as the time from the date of diagnosis until death or last contact. Survival analysis was performed using the Kaplan-Meier method, and the difference between the outcomes was evaluated by the log-rank test. Further, the effect of different predictor variables on overall survival was assessed using univariate and multivariate Cox regression analysis. A P-value of less than 0.05 was considered statistically significant. All the statistical analyses were performed using the IBM SPSS version 23.0 software (SPSS Inc., Chicago, IL, USA).

## Results

**Figure 1:**
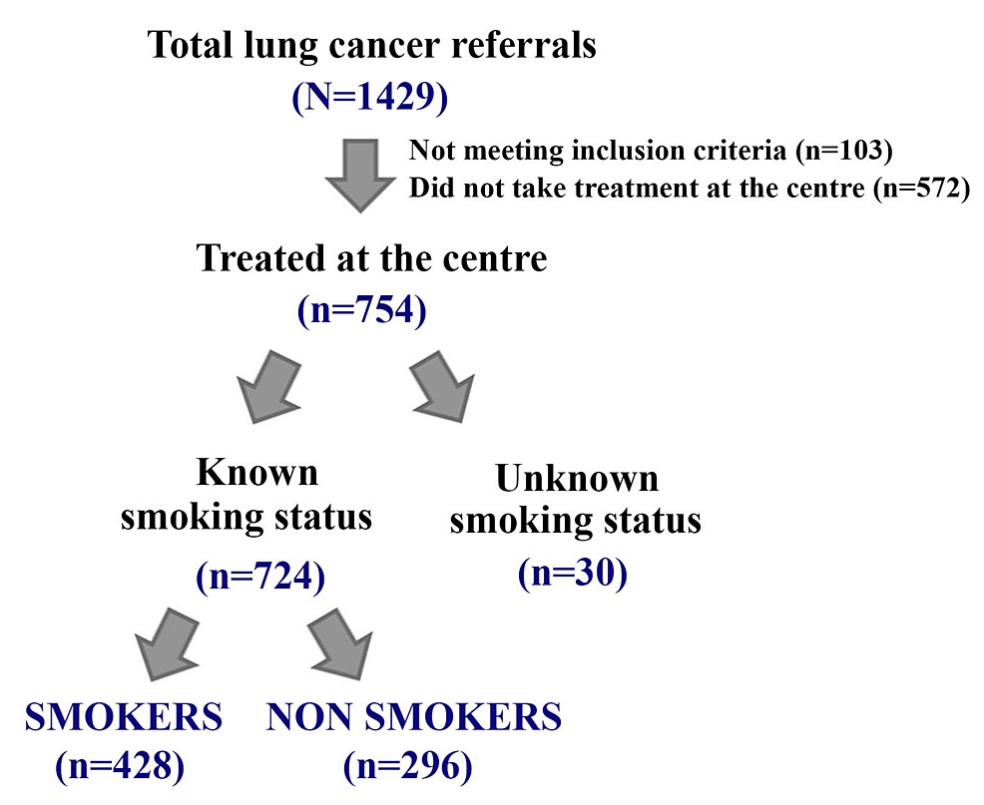
Flow diagram of the study group (2012-2014).

Smoking status was determined for 724 of the 754 lung cancer patients treated at the center. The remaining 30 patients were either reluctant to share their smoking habits or could not be contacted. Therefore, we analysed the data of the cohort of 724 patients for whom complete treatment and smoking history could be ascertained. A flow diagram of the study is shown in Figure 1.

Patient demographics are summarised in Table 1. Smokers comprised 59.1% (428/724) and non-smokers 40.9% (296/724) of the study sample. The median age of the study cohort was 60 years (range: 25-90 years), with the median age of non-smokers being significantly less than that of smokers (58 vs 61 years; P = 0.0004). Non-smokers presented with the disease at a younger age (<40 years) compared to smokers, in whom the disease tended to present at a later age (>60 years) (P = 0.004).

Males constituted about three-fourths of the study cohort. There were comparatively fewer females than males who smoked (7% vs 93%). A significant difference was noted in smoking habits with respect to the patients' occupations (P < 0.001) (Table 1); specifically, smoking was common among farmers and the retired or dependent group and less prevalent among housewives.

**Table 1:** Patient demographics (N = 724, Lung cancer cases).

	Total	Smokers	Non-smokers	P value
	N = 724 (%)	n = 428 (%)	n = 296 (%)	
Age (years)				
Median (Range)	60 (25-90)	61 (25-90)	58 (25-90)	0.0004
< 40	36 (5)	13 (3)	23 (7.8)	0.004
40-60	350 (48.3)	200 (46.7)	150 (50.7)	
> 60	338 (46.7)	215 (50.2)	123 (41.6)	
Gender				
Male	547 (75.6)	398 (93)	149 (50.3)	<0.001
Female	177 (24.4)	30 (7)	147 (49.7)	
Region of residence in India				
North	615 (84.9)	367 (85.7)	248 (83.8)	0.84
East	87 (12)	49 (11.4)	38 (12.8)	
Central	8 (1.1)	5 (1.2)	3 (1)	
West	14 (1.9)	7 (1.6)	7 (2.4)	
Occupational history				
Business	68 (9.4)	45 (10.5)	23 (7.8)	<0.001
Employed	182 (25.1)	109 (25.5)	73 (24.7)	
Farmer	64 (8.8)	50 (11.7)	14 (4.7)	
Housewife	138 (19.1)	24 (5.6)	114 (38.5)	
Labour	4 (0.6)	3 (0.7)	1 (0.3)	
Retired/dependent	266 (36.7)	197 (46)	69 (23.3)	
Student	2 (0.3)	0 (0)	2 (0.7)	

The comparison of clinical characteristics between the smoking and non-smoking groups is presented in Table 2. Cough and dyspnea were the most common initial symptoms in both smoking and non-smoking groups. Smokers were diagnosed with chest pain (24.1% vs 14.5%, P = 0.002) and hemoptysis (15.2% vs 8.8%, P = 0.01) more frequently than non-smokers, who were more often diagnosed with back pain (12.8% vs 7.0%, P = 0.01) and neurological conditions (8.8% vs 4.9%, P = 0.04). The majority of the cases presented at advanced disease stages, with 96.4% presenting at stage III or IV. A significant difference was noted upon comparing the stage of disease presentation between smokers and non-smokers (P < 0.001), as more non-smokers presented the disease at metastasis (89.2% vs 78.3%, P < 0.001). Bone and the brain were the more common metastatic sites among non-smokers (Table 2). Histologically, adenocarcinoma was the most frequently occurring subtype (56.6%), followed by squamous (27.2%) and small cell subtypes (10.4%). Tumor histology showed significant differences between smokers and non-smokers (P < 0.001), with non-smokers being more likely to be diagnosed with adenocarcinoma (77.4%) while squamous and small cell histologies were more common among smokers (37.6% and 13.8%, respectively). Concerning comorbidities, hypertension (15.5%) and diabetes (12.7%) were the most frequent (Table 2). Notably, compared to non-smokers, significantly more smokers had chronic obstructive pulmonary disease (COPD) as a comorbid condition (2.6% vs 0.3%, P = 0.03). The majority of patients received treatment with a palliative intent (81.6% of the cases). A total of 128 (17.7%) advanced lung cancer patients were offered an oral tyrosine kinase inhibitor, which was prescribed more often to non-smokers than smokers (28.4% vs 10.3%, P < 0.001).

**Table 2:** Clinical characteristics of the study group (N = 724, Lung cancer cases). *Others include adenoid cystic carcinoma, carcinoid tumors, poorly differentiated tumors, sarcomatoid carcinoma, and spindle cell carcinoma histologies. NOS: Not otherwise specified; COPD: Chronic obstructive pulmonary disease; CT: Chemotherapy; NACT: Neoadjuvant chemotherapy; RT: Radiotherapy; TT: Targeted therapy; IT: Immunotherapy

	Total	Smokers	Non-smokers	P-value
	N = 724 (%)	n = 428 (%)	n = 296 (%)	
Presenting symptom				
Asthenia	29 (4)	17 (4)	12 (4.1)	0.95
Back pain	68 (9.4)	30 (7)	38 (12.8)	0.01
Chest pain	146 (20.2)	103 (24.1)	43 (14.5)	0.002
Cough	328 (45.3)	206 (48.1)	122 (41.2)	0.07
Dyspnea	206 (28.5)	119 (27.8)	87 (29.4)	0.64
Fever	63 (8.7)	36 (8.4)	27 (9.1)	0.74
Haemoptysis	91 (12.6)	65 (15.2)	26 (8.8)	0.01
Hoarseness of voice	33 (4.6)	21 (4.9)	12 (4.1)	0.59
Loss of appetite	29 (4)	19 (4.4)	10 (3.4)	0.47
Loss of weight	69 (9.5)	41 (9.6)	28 (9.5)	0.96
Neurological symptoms	47 (6.5)	21 (4.9)	26 (8.8)	0.04
Systemic symptoms	132 (18.2)	76 (17.8)	56 (18.9)	0.69
Clinical stage				
I/II	26 (3.6)	17 (4)	9 (3)	<0.001
III	99 (13.7)	76 (17.8)	23 (7.8)	
IV	599 (82.7)	335 (78.3)	264 (89.2)	
Metastasis				
Absent	125 (17.3)	93 (21.7)	32 (10.8)	<0.001
Present	599 (82.7)	335 (78.3)	264 (89.2)	
Site of metastasis				
Adrenal	56 (7.7)	32 (7.5)	24 (8.1)	0.75
Bone	245 (33.8)	125 (29.2)	120 (40.5)	0.002
Brain	159 (22)	80 (18.7)	79 (26.7)	0.01
Liver	98 (13.5)	59 (13.8)	39 (13.2)	0.81
Pancreas	2 (0.3)	2 (0.5)	0 (0)	0.52
Pericardium	13 (1.8)	7 (1.6)	6 (2)	0.70
Pleural	201 (27.8)	117 (27.3)	84 (28.4)	0.76
Renal	2 (0.3)	1 (0.2)	1 (0.3)	1
Thyroid	1 (0.1)	1 (0.2)	0 (0)	1
Histopathology				
Adenocarcinoma	410 (56.6)	181 (42.3)	229 (77.4)	<0.001
Adenosquamous	4 (0.6)	3 (0.7)	1 (0.3)	
Squamous	197 (27.2)	161 (37.6)	36 (12.2)	
Small cell	75 (10.4)	59 (13.8)	16 (5.4)	
Large cell	6 (0.8)	5 (1.2)	1 (0.3)	
NSCLC (NOS)	9 (1.2)	5 (1.2)	4 (1.4)	
*Others	23 (3.2)	14 (3.3)	9 (3.0)	
Comorbidity				
Chronic kidney disease	3 (0.4)	1 (0.2)	2 (0.7)	0.57
COPD	12 (1.7)	11 (2.6)	1 (0.3)	0.03
Diabetes	92 (12.7)	55 (12.9)	37 (12.5)	0.89
Hypertension	112 (15.5)	67 (15.7)	45 (15.2)	0.87
Ischaemic heart disease	2 (0.5)	0 (0)	2 (0.3)	0.52
Stroke	5 (0.7)	5 (1.2)	0 (0)	0.08
Treatment				
Surgery	24 (3.3)	13 (3)	11 (3.7)	0.62
Surgery + Adjuvant CT	10 (1.4)	7 (1.6)	3 (1)	0.54
NACT	45 (6.2)	32 (7.5)	13 (4.4)	0.09
CT+RT	19 (2.6)	15 (3.5)	4 (1.4)	0.07
Palliative (CT and/or RT)	591 (81.6)	352 (82.2)	239 (80.7)	0.61
TT	128 (17.7)	44 (10.3)	84 (28.4)	<0.001
IT	3 (0.4)	1 (0.2)	2 (0.7)	0.57
Supportive care	69 (9.5)	38 (8.9)	31 (10.5)	0.47

**Figure 2:**
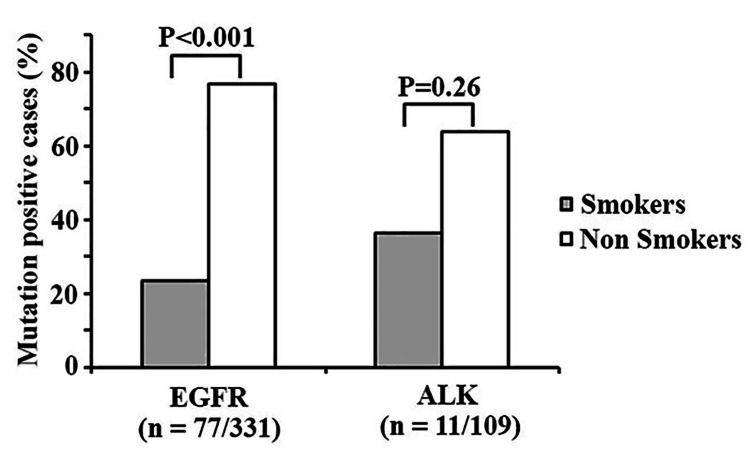
Frequency of EGFR and ALK genomic alterations in the tested cases compared according to the smoking status.

A total of 331 cases were tested for EGFR mutation, of which 77 (23.3%) were positive. A significant difference was noted for the EGFR mutation among smokers and non-smokers, as in comparison to smokers, more non-smokers harbored the EGFR mutation (59/77, 76.6% vs 18/77, 23.4%; P < 0.001) (Figure 2). The ALK mutation was assessed in 109 cases; among them, 11 patients (10.1%) tested positive. The non-smokers harbored the ALK mutation more than the smokers (7/11, 63.6 vs 4/11, 36.4%), but the difference did not achieve statistical significance (Figure 2).

**Figure 3:**
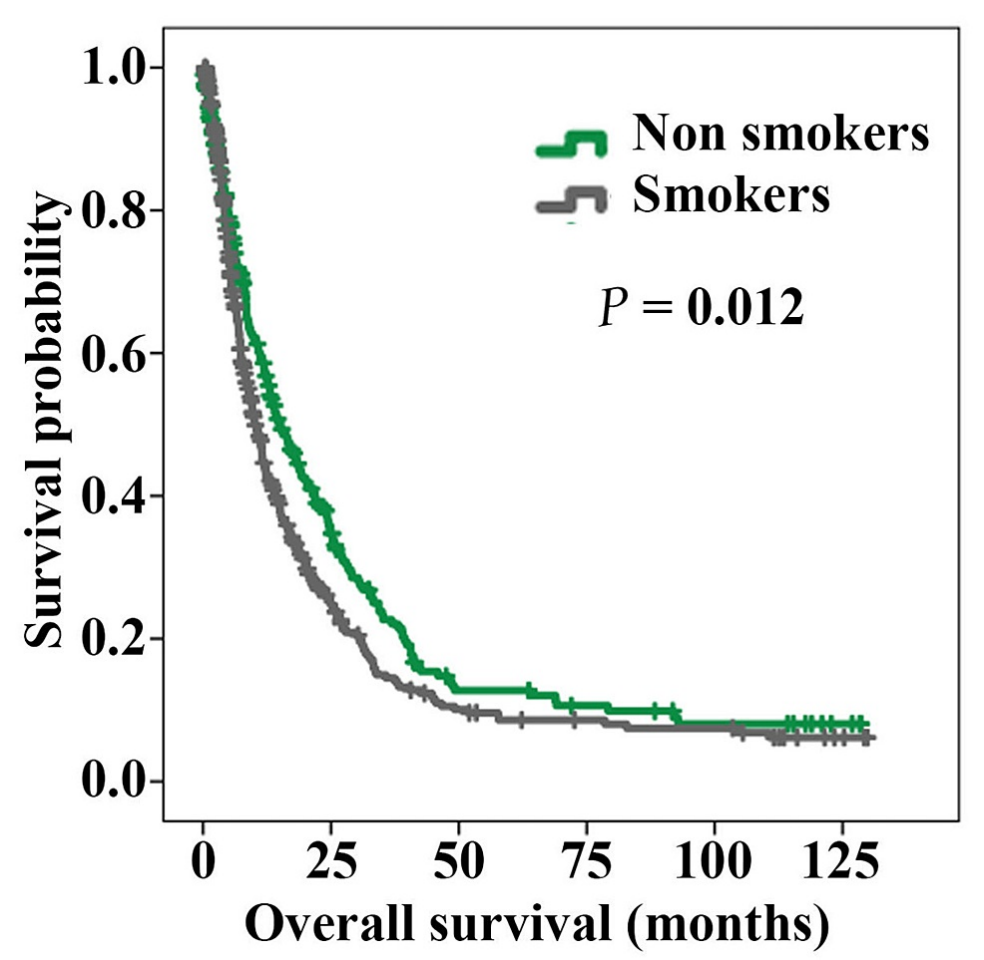
Kaplan-Meier curves comparing overall survival according to smoking status (Smokers: 428 cases, Non-smokers: 296 cases; lung cancer patients).

The overall survival of the cohort was 7% at 129 months; the median duration of survival was 11.9 months (95% CI: 10.33-13.47 months). In 71.3% of the cases, follow-up information was available. Kaplan-Meir analysis with respect to smoking status showed a significant difference between the smokers and non-smokers (P = 0.012) (Figure 3). The overall survival was inferior in the smoking group (median overall survival: 10.17 months; 95% CI: 8.75-11.58) as compared to the non-smoking group (median overall survival: 15.13 months; 95% CI: 11.82-18.45).

Next, we examined the variables associated with survival. Through univariate Cox regression analysis, we found that older age, having tumor histology other than adenocarcinoma, a higher disease stage, and having a smoking history significantly increased the hazards of death (Table 3), while the patients who received targeted therapy or immunotherapy were associated with a decreased hazard of death (Table 3). Through multivariate Cox regression analysis, younger age, adenocarcinoma histology, early-stage disease, and antitumor treatment with targeted or immunotherapy were the independent favourable prognostic variables for survival in the cohort (Table 3).

**Table 3:** Cox regression analysis of the predictors related to overall survival in the study cohort (N = 724 lung cancer patients). *Age as a continuous variable. Reference variables: Male gender; no comorbidity; adenocarcinoma histology; stage I/II; non-smokers; not received targeted/ immunotherapy. HR = Hazard ratio, CI = Confidence interval

	Univariate Cox regression			Multivariate Cox regression		
Predictor variable	HR	95% CI	P-value	HR	95% CI	P-value
Female	0.84	0.69-1.04	0.11			
Presence of comorbidity	0.97	0.79-1.20	0.81			
*Age	1.02	1.007-1.02	<0.001	1.02	1.01-1.02	<0.001
Non-adenocarcinoma histology	1.23	1.03-1.47	0.023	1.22	1.01-1.49	0.04
Stage III	2.17	1.12-4.26	0.024	2.39	1.22-4.71	0.01
Stage IV	4.32	2.30-8.12	<0.001	5.35	2.83-10.10	<0.001
Smokers	1.26	1.05-1.51	0.01	1.18	0.97-1.45	0.09
Received targeted therapy/ immunotherapy	0.8	0.64-0.99	0.04	0.75	0.59-0.95	0.02

## Discussion

Lung cancer has long been considered a disease of smokers. However, this notion is undergoing a paradigm shift due to an increase in the proportion of non-smoking lung cancer patients. Currently, in India, non-smoking lung cancer cases are quite common, yet they go understudied in most reports. It is important to research lung cancer in non-smokers because this subset of patients has unique epidemiology, clinicopathology, genetic makeup, treatment response, and survival characteristics. This study delves into the count, clinicopathological features, and outcome of non-smoking lung cancer patients treated at a tertiary cancer centre in North India.

The frequency of non-smokers in our study was around 41%, which is high when compared to the reports from developed western countries but parallels other studies from India [3,6,7,18]. There have been wide variations in the reported proportion of non-smoking lung cancer patients, with higher rates in the Asian cohort [6,9,19]. Paradoxically, the prevalence of smoking and poor air quality is higher in developing countries, yet more than 30% of lung cancer cases in Asia are never smokers, whereas only 10-15% of cases have been reported in Western literature [11,20,21]. Cultural and ethnic differences, together with genetic predisposition, could be the major reasons for such variability. In this study, the majority of cases (78%) were from urban Delhi and adjoining regions, which are constantly exposed to air pollutants and secondhand smoke from vehicles, and therefore, the potential contribution of the environment towards the development of lung cancer in the non-smoking patients cannot be negated [22,23].

We observed significant differences between smokers and non-smokers regarding gender, age at disease onset, presenting symptoms, clinical stage, tumor histology, and comorbidities. Non-smoking status was associated with younger patients, metastatic disease, and adenocarcinoma histology. Consistent with other Asian studies, the median age of non-smokers with lung cancer was lower than that of smokers [4,6,12]. Differing reports have emerged from Western countries where cancer in non-smokers presented similarly or at an older age than in smokers [10,24,25]. This could be due to other differential risk factors other than smoking or the late age of smoking initiation in Asia when compared to their Western counterparts [6,26]. The late presentation of disease in the non-smokers in our study could be due to a comparatively lesser degree of clinical suspicion of lung cancer in non-smokers than in smokers. Another reason for late diagnosis in non-smokers could be that the majority of non-smokers had adenocarcinoma histology, which is described as having a peripheral origin and symptoms manifesting at a later stage of disease development [27]. Squamous cell lung cancer, however, is more often central, grows near the epithelial airways, and leads to early symptoms [27] such as hemoptysis, which was a common symptom in our smoking patients. Regarding tumor genomic alterations, the EGFR mutations were detected in 23.3% of the patients, and as expected, they were more prevalent in non-smokers than in smokers. These findings are analogous to previously published reports from Asia that report a higher frequency of EGFR mutations in Asian cohorts [18,25,28].

Generally, the survival rate for lung cancer is poor, and most reports suggest survival rates lower than 16% [15,18]. In our study, the 10-year survival rate was 7%, and in line with previous reports, the non-smokers exhibited significantly better survival than the smokers [10,25,29]. Despite late-stage presentation, young age, fewer comorbid conditions, and targeted therapy, these factors could have positively contributed to improved survival in the non-smoking lung cancer patients in our cohort.

There were only 30 (7%) women in the smoking group, which could be because of cultural reasons, as, in India, women are generally discouraged from smoking. Among female lung cancer patients, 83% did not smoke. The effects of hormonal predisposition and environmental exposure to carcinogens such as cooking fumes cannot be ruled out for these patients. Although we have not studied these factors in detail, they are worth investigating in future studies.

The limitation of the study is its retrospective nature, which makes it difficult to seek causal associations. About 28.7% of the cases were lost in the follow-up stage. Moreover, the study is tertiary cancer centre-based and is bound to show more advanced cases. The strength of this study is the long-term follow-up of the patients and its relatively large sample size.

## Conclusions

More than one-third of the lung cancer patients in our study were non-smokers, in whom cancer tends to present at an early age and at an advanced stage, and who have adenocarcinoma histology. The frequency of driver gene mutations in EGFR and ALK genomic rearrangements was high in non-smokers, and their survival was found to be longer than that of smokers. In our cohort, the non-smokers appear to be a targetable group with better survival prospects than the smokers.
